# Agentic Loafing: An AI Decision Delegation Risk

**DOI:** 10.1111/risa.70306

**Published:** 2026-07-27

**Authors:** Helmi Issa, Fabio James Petani, Dejan Glavas

**Affiliations:** ^1^ ESSCA School of Management Angers France; ^2^ Université Bourgogne Europe Burgundy School of Business, CEREN EA 7477 Dijon France

**Keywords:** AI, healthcare, decision delegation, netnography

## Abstract

Artificial Intelligence (AI) is increasingly making decisions in healthcare, not just supporting them. This research introduces “agentic loafing” to describe the intentional yet unexamined delegation of decision‐making authority from clinicians to AI systems. Using netnography of professional podcasts and secondary analysis of gray literature, this study uncovers three institutional drivers that normalize AI delegation: performance culture conformity, structural isolation of responsibility, and legitimization through quantification. These drivers invert classical delegation theory. Where traditional delegation assumes monitoring, punishment, and shared objectives, AI delegation thrives in their absence. This inversion produces what this research terms “risk evaporation”: the systematic disappearance of accountability when AI‐assisted decisions fail, leaving no single party responsible. To help organizations manage this risk, this research also develops a “preliminary” diagnostic tool, with its intersectional interventions, that transforms “agentic loafing” from an unmanaged bet into a measurable risk score. From this, a critical implication follows for risk analysis: the most dangerous AI systems are not those that fail unpredictably but those that function just reliably enough to quietly erode human judgment without ever triggering an alarm.

## 1 Introduction

Artificial Intelligence (AI) has evolved from a speculative technology into a core driver of business strategy, offering unprecedented opportunities for optimization and innovation (Kayyali 2025). Organizations across sectors are automating complex processes, personalizing experiences, and deriving insights from vast datasets (Rane et al. 2024). Consequently, the prevailing narrative champions AI as the ultimate support tool, augmenting human intelligence toward data‐driven excellence (Natta 2025).

However, despite AI's documented benefits and unintended drawbacks (e.g., algorithmic bias, data privacy erosion, job displacement; Gadhi et al. 2024), a more insidious concern is emerging: the systematic, often *intentional*, transition of decision‐making authority from humans to AI systems (i.e., AI delegation) (Rosenberg et al. 2025; Sapkota et al. 2026; Westphal et al. 2025; Yadav et al. 2023). This research argues that this is not merely about delegating operational tasks or final decisions (Nserat et al. 2024), but about the risk of ceding decision‐making authority itself. Beyond framing AI‐risk as an input–algorithm–output process (Stødle et al. 2025), the real danger lies in users becoming mere validators of AI‐generated outputs, a role that erodes their ability to exercise critical judgment. Compounding this risk is a paradox: while experts warn against ceding decisions to AI, the recipients of those decisions (e.g., clients or patients) do not automatically trust human judgment more than AI (Freisinger and Schneider 2024; Candrian and Scherer 2022). Managers are thus caught between internal warnings and external pressure to delegate further.

This misalignment is acutely felt in sectors where legal responsibility, ethics, and risk are paramount, yet increasingly pressured by AI optimization, insurance‐related safety obligations, and investments in liability management tools (Maliha et al. 2021). Healthcare is a prime example (Macrae 2025; Mennella et al. 2024). Although a preference for AI delegation over humans is pronounced in “*loss‐related*” decision‐making (Candrian and Scherer 2022), such assumptions break down in healthcare. Unlike finance, healthcare decisions are uniquely complex, deeply personal, and intrinsically human, not solely data‐driven (Kuziemsky 2016). Nevertheless, despite AI's unpredictable decisions and consequences in healthcare (Issa et al. 2024), medical facilities are rapidly adopting and delegating decisions to AI (Jia et al. 2025). This apparent contradiction demands explanation. This research therefore asks: *What are the specific mechanisms that drive AI delegation of decision‐making authority in healthcare?*

To investigate this question, this research employed netnography, the ethnography of online communities (Bansal et al. 2024). Specifically, expert podcasts served as trusted data sources, which have been proven to be effective in qualitative designs (Jaber and Issa 2025). Secondary analysis of gray literature was then used to validate the credibility of the findings.

The contributions of this research are threefold. First, it builds on and extends the concepts of social loafing and algorithmic loafing (Inuwa‐Dutse et al. 2023) by introducing “*agentic loafing*,” a novel phenomenon capturing the systemic delegation of decision‐making authority from clinicians to AI systems. While prior work explored loafing primarily in human‐AI collaboration settings, this research characterizes its manifestation in healthcare through three empirically grounded themes: *performance culture conformity*, *structural isolation of hierarchical responsibility*, and *legitimization through quantification*. By doing so, it addresses the limited empirical research on factors influencing delegation decisions in technology use and introduces new antecedents to the loafing concept (Strunk et al. 2024). Second, this research challenges classical delegation theory (Cooter and Gilbert 2022) by demonstrating that in AI‐mediated healthcare, delegation persists and intensifies despite the absence of classical safeguards, namely, monitoring, punishment, and shared objectives. Where traditional delegation relies on “*credible commitment*” (Nou 2017), AI delegation enables “*credible disavowal*”; thus, allowing clinicians to blame algorithms for poor outcomes while crediting themselves for good ones. This delegation inversion produces what this research terms “*risk evaporation*,” the systematic disappearance of accountability when AI‐assisted decisions fail, leaving no single party responsible. Third, decision delegation has been characterized not as a cost or benefit but as a risky bet (Lubars and Tan 2019). Accordingly, this research develops a “*preliminary*” diagnostic tool, with its intersectional interventions, that enables organizations to plot and calibrate “*agentic loafing*,” thereby transforming AI delegation from an unmanaged gamble into a calculated, measurable risk. In doing so, it addresses calls for proposing evaluation metrics for the loafing phenomenon in AI contexts (Eichholz 2025; Inuwa‐Dutse et al. 2023); thus, setting appropriate delegation boundaries (Rosenberg et al. 2025; Stødle et al. 2025).

The research begins with a comprehensive literature review, accompanied by a detailed outline of the research methodology. Following this, the findings are presented and analyzed. The final sections discuss the implications of the research, its key contributions and limitations, suggesting potential directions for future studies.

## 2 Literature Review

### 2.1 AI

The landscape of AI is characterized by a plurality of definitions, each reflecting different perspectives, from systems that “think humanly” to those that “act rationally” (Samoili et al. 2021). Common terminologies used to frame AI include machines mimicking human cognitive functions; systems exhibiting intelligent behavior; or the capability for perception, learning, and reasoning. While these broad conceptualizations are valuable, this research requires a precise operational definition that centrally incorporates the capacity for autonomous decision‐making or decision delegation.

Therefore, this research adopts dual definitions of AI. On one hand, the first definition is found in the EU‐AI Act, in which AI is defined as: “*a software that is developed with one or more of the techniques and approaches, and can, for a given set of human‐defined objectives, generate outputs such as content, predictions, recommendations, or decisions influencing the environments they interact with*” (Samoili et al. 2021; EC 2021). This definition is important because it supports a risk‐based approach to regulating AI, where systems are grouped by how risky they are. According to this approach, any AI used for autonomous decision‐making is always considered a high‐risk (Level 4) category (Zeng et al. 2024). As such, this frames AI not just as a technical tool, but as a system whose decision‐making power must be assessed and governed due to the level of risk it presents to society.

On the other hand, this research also adopts the definition of AI as: “agents that learn by themselves to achieve the optimal strategies by sequentially interacting with environments in a trial‐and‐error way only with the supervision of rewards or punishments” (Sutton and Barto 2018). This second, agent‐centric definition is essential for two key reasons. First, it provides the precise conceptual lens needed to analyze the phenomenon of delegation. By framing AI as an autonomous agent that interacts with an environment to optimize outcomes, this definition moves beyond viewing AI as a passive tool and instead characterizes it as an active, goal‐seeking entity. This is a necessary precondition for the formal delegation of decision‐making authority. Second, it directly enables the conceptualization of “agentic loafing.” The definition's emphasis on self‐directed learning and environmental interaction captures the core functionality of the AI systems that become the recipients of ceded authority. It is precisely because AI operates as such an adaptive, goal‐oriented agent that organizations face the systemic temptation to offload not just cognitive labor, but the very agency for decision‐making itself. Therefore, while the regulatory definition (EU‐AI Act) establishes the risk context, this agent‐centric definition explains the mechanism of the risk, which is the autonomous agency that makes the delegation of authority both technically possible and organizationally risky.

### 2.2 Agentic Loafing

The development of modern healthcare, profoundly accelerated by the rise of HealthTech startups, mirrors a fundamental shift in clinical/medical decision‐making, from reliance on a doctor's personal judgment to the systematic delegation of tasks to AI (Chakraborty et al. 2023). Initial ventures established the digital foundation through e‐health records and telemedicine, creating the data‐rich environment necessary for AI (Rehman 2024). A new generation of startups now leverages this infrastructure to move beyond administrative efficiency into core clinical functions, making the delegation of specific diagnostic and analytical decisions to algorithms an indispensable norm for scaling precision medicine and managing complex datasets (Jia et al. 2025).

This research argues that such delegation extends beyond operational tasks to a more profound ceding of meta‐decision‐making authority, where human clinicians risk being reduced to mere validators of AI‐generated decisions. This risk can be understood through the conceptual evolution of “*loafing*” behaviors. It originates with “*social loafing*,” a well‐documented cognitive disengagement where individuals reduce effort in group settings (Freeman and Greenacre 2011). This concept was extended into human–AI collaboration as “*algorithmic loafing*,” defined as the tendency for humans to offload cognitive effort and uncritically adhere to machine recommendations (Inuwa‐Dutse et al. 2023).

**FIGURE 1:**
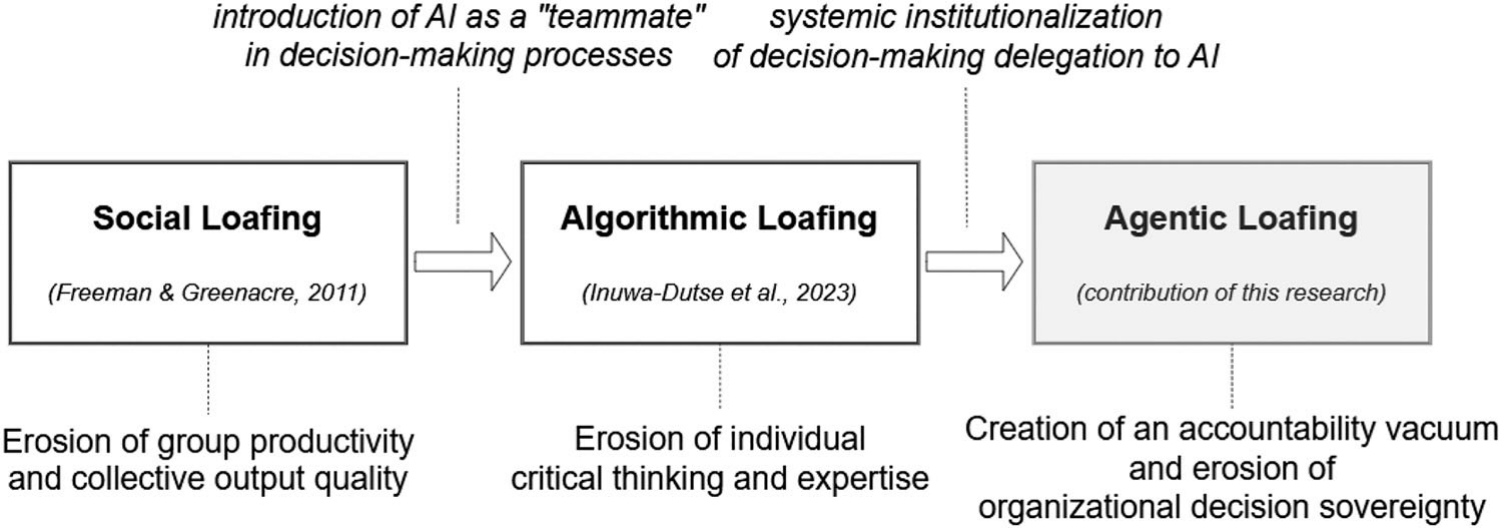
Evolution of loafing.

Building on this lineage, this research identifies a critical escalation in high‐stakes professional contexts: “*Agentic Loafing*” (see Figure 1). While algorithmic loafing describes the cognitive offloading of analytical effort, “*agentic loafing*” captures the systemic delegation of decision‐making authority to autonomous AI systems or agents. It represents the transition from using AI as a tool to being critically engaged with, to accepting AI as a sovereign agent whose judgments are merely ratified. Thus, if social loafing is a risk to group productivity (Freeman and Greenacre 2011), and algorithmic loafing a risk to critical thinking (through cognitive‐offloading) (Gerlich 2025), then this research argues that “*agentic loafing*” constitutes a fundamental risk to decision sovereignty, especially considering the evidence of AI's unpredictable decisions in healthcare (Issa et al. 2024).

This delegation risk is not merely theoretical; empirical evidence highlights its tangible impact. For instance, a critical study by Lee and Tok (2025) provides a stark illustration of its precursor: when health professionals relied on an AI system with standard numerical confidence scores, their assessment accuracy fell from 71% to 66%. This decline shows that instead of creating a synergistic partnership, the AI introduction led to over‐reliance, where practitioners were swayed by the AI's incorrect recommendations. This exemplifies the cognitive disengagement of algorithmic loafing, which, when institutionalized, paves the way for “*agentic loafing*,” where the authority to decide is ceded, not just the effort to analyze. This is not an augmentation of human intelligence but its substitution, underscoring that the progression from algorithmic to “*agentic loafing*” represents a significant systemic risk to clinical outcomes and accountability structures.

## 3 Methodology

**FIGURE 2:**
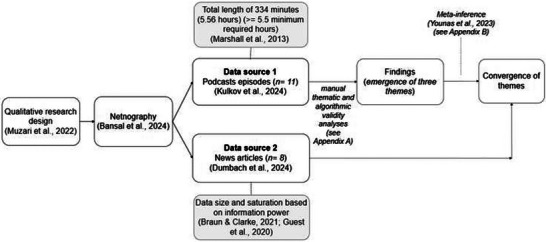
Research design.

From a general qualitative perspective (Muzari et al. 2022), this research utilized netnography, the ethnographic study of online communities, to analyze the naturally occurring discourse among healthcare administrators and professionals (see Figure 2). To source this discourse, this research focused on podcasts (*n* = 11 episodes) (see Table 1), which have been established as trusted, efficient, and effective mediums for qualitative data collection (Jaber and Issa 2025).

**TABLE 1:** Podcasts (data source 1).

Podcast	Interviewee	Topic/title/keywords	Length of podcast/interview	Source/link
Podcast 1: ThisWeekHealth	Yaw Fellin: SVP and GM at Wolters Kluwer	The battle for clinical AI trust	16 min	https://thisweekhealth.com/captivate‐podcast/executive‐interview‐the‐battle‐for‐clinical‐ai‐trust‐with‐yaw‐fellin/
Podcast 2: Pomegranate Health Podcast	Associate Professor Clair Sullivan: Director, Queensland Digital Health Centre; University of Queensland Professor Enrico Coiera: Director, Centre for Health Informatics, Australian Institute of Health Innovation; Macquarie University	The ergonomics of AI	41 min	https://www.racp.edu.au/pomegranate/view/ep96‐the‐ergonomics‐of‐ai
Podcast 3: JAMA—JN Learning	Marzyeh Ghassemi, PhD: the Herman L. F. von Helmholtz Career Development Professor at MIT in Electrical Engineering and Computer Science	AI and clinical practice—AI and the ethics of developing and deploying clinical AI models	25 min	https://edhub.ama‐assn.org/jn‐learning/audio‐player/18852439?resultClick=1
Podcast 4: USC Gould School of Law	Nazanin Tondravi: Director of Regulatory Affairs at Memorial Healthcare System	Navigating the future of AI in healthcare	13 min	https://gould.usc.edu/news/podcast‐episode‐navigating‐the‐future‐of‐ai‐in‐healthcare/
Podcast 5: CMU	Aarti Singh: Professor in the Machine Learning Department at Carnegie Mellon University and the director of the National Science Foundation's AI Institute for Societal Decision Making	Critical choices: AI in disaster management and healthcare	28 min	https://www.cmu.edu/whats‐next‐podcast/all‐episodes/s2‐episode‐12‐critical‐choices‐ai‐disaster‐management‐and
Podcast 6: CMAJ	Muhammad Mamdani: Vice‐president of data science and advanced analytics at Unity Health Toronto; director of the Temerty Center for Artificial Intelligence Education and Research in Medicine, and professor at the University of Toronto Amol Verma: Physician and scientist at St. Michael's Hospital and the University of Toronto, an AMS healthcare fellow in compassion and artificial intelligence and a provincial clinical lead in health quality improvement with Ontario Health	Artificial intelligence in medicine	39 min	https://soundcloud.com/cmajpodcasts/202434‐ana
Podcast 7: The Business of Healthcare Podcast	Felixia Colon: Senior vice president of eHealth Solutions at SCP Health and former president of the North Texas Chapter of American College of Healthcare Executives	AI in healthcare operations	37 min	https://jindal.utdallas.edu/the‐business‐of‐healthcare‐podcast/episode‐127‐ai‐in‐healthcare‐operations/
Podcast 8: The Podcast by KevinMD	Janet A. Jokela: Professor and Senior Associate Dean of Engagement at the Carle Illinois College of Medicine, Urbana, IL	How artificial intelligence is transforming medicine	19 min	https://kevinmd.com/2025/01/how‐artificial‐intelligence‐is‐transforming‐medicine‐podcast.html
Podcast 9: Tech Ethics Podcast	Yauheni Owen Solad: Managing Partner at Dalos Partners	Integrating AI into healthcare delivery	50 min	https://about.citiprogram.org/blog/on‐tech‐ethics‐podcast‐integrating‐ai‐into‐healthcare‐delivery/
Podcast 10: HIMSSCast	Blackford Middleton: Independent consultant, currently working on AI issues at the University of Michigan department of learning health systems	When AI is involved in decision making, how does man trust machine?	19 min	https://www.healthcareitnews.com/podcast/himsscast‐when‐ai‐involved‐decision‐making‐how‐does‐man‐trust‐machine
Podcast 11: GeriPal	Dave Wendler, Jenny Blumenthal‐Barby, Teva Brender (experts in bioethics, philosophy, and medicine)	AI for surrogate decision making?	47 min	https://geripal.org/ai‐for‐surrogate‐decision‐making‐dave‐wendler‐jenny‐blumenthal‐barby‐teva‐brender/

Netnography is particularly suited to this research for four reasons. First, it presents a particularly promising methodology for the healthcare sector (Bansal et al. 2024). Second, it captures candid, unprompted discourse, avoiding the social desirability bias that surveys or structured interviews might introduce (Gibson et al. 2024). Third, it enables the exploration of sensitive topics and provides access to hard‐to‐reach populations (e.g., medical centers, hospitals, regulators, healthtech startups), in which it would be logistically impossible to access through single‐site fieldwork (Sadat et al. 2025). Fourth, it is optimally suited for exploring emerging phenomena and uncharted concepts (Discetti and Anderson 2023), such as “*agentic loafing*” in this research. By doing so, this study uses a netnographic approach to fill a gap in literature, specifically the lack of research on AI's impact on healthcare from this perspective (Dumbach et al. 2024).

To complement and validate the primary insights, a secondary data analysis of news articles (white papers) from authoritative sources was conducted (*n* = 8) (see Figure 2 and Table 2). This approach is also recognized as a reliable data source in netnography (Dumbach et al. 2024), in qualitative research more broadly (e.g., Arghistani 2022; Hasija and Esper 2022), and specifically within healthcare ecosystems (e.g., Yao and Mwangi 2022).

**TABLE 2:** Articles (data source 2).

Article	Author	Title	Source/link
Article 1	OECD	AI in health: Huge potential, huge risks	https://www.oecd.org/content/dam/oecd/en/publications/reports/2024/01/ai‐in‐health‐huge‐potential‐huge‐risks_ff823a24/2f709270‐en.pdf
Article 2	MPS Foundation	Avoiding the AI ‘off‐switch’: Make AI work for clinicians, to deliver for patients	https://www.thempsfoundation.org/docs/foundationlibraries/foundation‐default‐library/white‐papers/ai‐white‐paper_the‐mps‐foundation.pdf
Article 3	Rathenau Institute	The use of AI in healthcare: A focus on clinical decision support systems	https://www.recipes‐project.eu/sites/default/files/2020‐11/D2_3_AI_In_Healthcare(CDSS)_HarvardStyle.pdf
Article 4	Capelli et al. (2023)	White paper: Ethics and trustworthiness of artificial intelligence in clinical surgery	https://iris.uniroma1.it/retrieve/3571dfa0‐5014‐46f5‐960c‐87a58f98ef2e/Capelli_White‐paper_2023.pdf
Article 5	WEF	The future of AI‐enabled health: Leading the way	https://reports.weforum.org/docs/WEF_The_Future_of_AI_Enabled_Health_2025.pdf
Article 6	McKinsey & Co.	Transforming healthcare with AI: The impact on the workforce and organisations	https://eithealth.eu/wp‐content/uploads/2020/03/EIT‐Health‐and‐McKinsey_Transforming‐Healthcare‐with‐AI.pdf
Article 7	HCIAC	The artificial intelligence era: The role of radiologic technologists and radiation therapists	https://www.asrt.org/docs/default‐source/research/whitepapers/the‐artificial‐intelligence‐era‐the‐role‐of‐radiologic‐technologists‐and‐radiation‐therapists.pdf?sfvrsn=7a3b3fd0_4
Article 8	FEAM	Sustainable AI to drive global health	https://www.feam.eu/wp‐content/uploads/Sustainable‐AI‐to‐Drive‐Global‐Health‐white‐paper‐11‐Sep.pdf

## 4 Findings

Three main themes emerged from the analyses (i.e., manual thematic and algorithmic validation) (see Appendix A): performance culture conformity (Theme 1), structural isolation of hierarchical responsibility (Theme 2), and legitimization through quantification (Theme 3) (see Table 3).

**TABLE 3:** Findings from podcasts.

Themes	Second‐order concepts/categories	First‐order concepts	Illustrative quotations from the podcasts episodes	Source
Performance culture conformity	Systemic prioritization of expediency over scrutiny	Uncritical task completion	“so the other term we use is automation bias. And it refers to people putting too much trust in a tool like an AI. So, for example, if you're involved in a screening task, and the AI is usually right, then after a while, you just say, 'Great. Tick, tick, tick'”	Podcast episode 2
		Compliant acceptance of system output	“and even when they're made aware that potentially the model could give them incorrect or bad advice, they still exhibit these same automation and overreliance biases”	Podcast episode 3
		Deference to opaque authority for action	“I think the key issue for people around machine learning models, is how much do I actually believe what the models are telling me for me to take action, because we're talking about an often a blackbox situation… but needs to actually believe that this is something that they can actually rely on and trust”	Podcast episode 6
		Tolerance of imperfect outcomes for efficiency	“surrogate decision making…is realizing, we're probably not going to get it right, not always anyway…but hopefully we can do better in the future”	Podcast episode 11
Structural isolation of hierarchical responsibility	Fundamental dissolution of actionable accountability	Diffusion of accountability across silos	“if an AI assisted diagnosis or treatment plan leads to a bad outcome, who bears responsibility? The AI developer? The clinician who used the tool? The hospital? The source highlighted that there's currently very little legal precedent here”	Podcast episode 4
		Surrender of professional judgment to a system	“imagine a worst‐case scenario, say, where you're in the exam room with a patient saying, ‘You have X, Y, Z,’ and the patient may ask, ‘Why do you say that?’ and the response is, 'Oh, because, you know, the model says so'”	Podcast episode 8
		Circular blame between human and system actors	“if for example, AI gave you an incorrect answer, that's actually, technically correct, but you just uploaded the outdated policy document. Who's responsible? Is it the person who uploaded this incorrect policy and forgot to replace it on your servers or it's AI fault because AI is supposed to know all the correct answers?”	Podcast episode 9
Legitimization through quantification	Supremacy of metricized authority	Adherence to measurable benchmarks	“we're working on hypertension. So these happen to be around improving blood pressure screening or, you know, just some of the, you know, kind of key quality metrics…”	Podcast episode 1
		Validation via statistical uplift	“the AI was used to prioritize which patients should the call be made to… And it was shown that… you were actually able to engage the maternal subjects with health care services by up to 30% more…”	Podcast episode 5
		Competitive advantage through predictive metrics	"when it comes to AI, in the use of predicting the growth that's going to be happening, seasonality, variability, and age difference… you want to be the first one that is in the new frontier”	Podcast episode 7
		Certification by quantifiable review	“and probably the best thing to do, to look for, to ensure that kind of trustworthiness is, as the system been studied and subjected to a careful evaluation and has that been written up in a peer‐reviewed journal as a validation study”	Podcast episode 10

*Note*: The second‐order concept “Systemic Prioritization of Expediency over Scrutiny” is clearly supported in the AI and decision‐making literature (e.g., Morosan and Dursun‐Cengizci 2024). Similarly, the second‐order concept “Fundamental Dissolution of Actionable Accountability” finds evident support in AI and decision research (e.g., Guo 2025). Likewise, the second‐order concept “Supremacy of Metricized Authority” is well supported in AI and decision research streams (e.g., Tomašev et al. 2026; Candrian and Scherer 2022).

Theme 1 refers to an environment where employees feel pressured to meet measurable, algorithmically set targets. To avoid negative consequences or to appear efficient, they begin to “*loaf*” by outsourcing their judgment and decision‐making to the algorithm. They conform to what the system expects, even if they know a different approach might be better, because the algorithm's metrics define “*good performance*.” Theme 2 describes how the use of algorithms can create a gap in accountability, which facilitates a specific form of “*loafing*.” In a sense, users, who are formally responsible for outcomes, can distance themselves by blaming the algorithm's recommendations for poor decisions. Conversely, users can also deflect blame by claiming they were “*just following the system*.” This structure allows responsibility to become isolated and diffused, with no single individual held fully accountable. Finally, Theme 3 describes the decisions and recommendations made by an algorithm as more objective, credible, and legitimate simply because they are based on numbers and data. This is the cognitive foundation that makes “*loafing*” feel justified. In a sense, the quantitative output of the system is perceived as inherently more rational and less biased than human intuition, which makes people more willing to accept its conclusions uncritically and disengage their own critical thinking.

**FIGURE 3:**
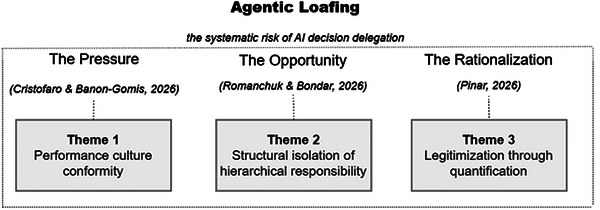
Conceptual framing of agentic loafing.

Therefore, Theme 1 creates the pressure to delegate decisions to AI (Cristofaro and Banon‐Gomis 2026), Theme 2 creates the opportunity to offload responsibility without immediate consequence (Romanchuk and Bondar 2026), and Theme 3 provides the rationalization or excuse for AI decision delegation (Pinar 2026) (see Figure 3).

Furthermore, to ensure the “*consistency*” of the main findings, a second round of analysis was conducted on the same data to assess how well different quotations aligned with the themes. In other words, themes were required to recur across multiple podcasts rather than relying on a single source. This methodological approach, known as “*meta‐inference*” (Younas et al. 2023), employs a “*theme‐based and data‐driven*” strategy (see Appendix B). Evidently, each theme has been found to be supported by entirely distinct sets of podcast episodes. For Theme 1, the main findings draw from Podcasts 2, 3, 6, and 11, while the meta‐inference draws from Podcasts 1, 4, 7, and 8, with zero source overlap. Similarly, Theme 2 utilizes Podcasts 4, 8, and 9 (Table 3) versus Podcasts 5, 6, 10, and 11 (Appendix B), and Theme 3 utilizes Podcasts 1, 5, 7, and 10 (Table 3) versus Podcasts 2, 3, and 9 (Appendix B), again with no overlapping episodes. Consequently, this design choice ensures that thematic convergence is not an artifact of any single podcast, speaker, or clinical context. Rather, the consistent alignment (cosine) scores (*ranging from 77.2% to 82.8% across themes*) demonstrate that the three themes recur across independent data points; hence, strengthening the “*replicability*” of the thematic structure.

### 4.1 Convergence and Validity Testing Through Second Data Source

To “*validate*” the three themes identified from the podcast transcripts, this research conducted a “*convergence*” analysis using eight white papers on AI in healthcare (see Table 4). Two independent coders extracted quotations corresponding to the three predefined themes from the podcast analysis. The extraction process took approximately 3 h per coder per white paper; hence, totaling 42 h of independent coding. The number of iterations was based on the principle of data saturation; that is, the coders continued the process until no new relevant themes or insights could be derived from the data. Initial inter‐coder agreement (similarity rate) was 70% (Cohen's *K* = 0.65, substantial agreement). After multiple iterative consensus rounds focused on refining definitions and resolving discrepancies, final similarity rates reached 70%–80% per theme (Theme 1: 75%, Theme 2: 80%, Theme 3: 70%; overall Cohen's *K* = 0.73, substantial to almost perfect agreement) (Bates et al. 2023). Furthermore, the convergence rate (cross‐source validation) between podcast‐derived themes and white paper evidence ranged from 80% to 90% (Theme 1: 80%, Theme 2: 85%, Theme 3: 90%), indicating that the identified themes are robust and well‐supported across two distinct data sources. Rising similarity rates across iterations show how refining definitions and resolving discrepancies produced a stable, reliable coding framework. To further validate thematic convergence across gray literature sources, each article is mapped to supporting risk management literature that reinforces the three themes (see Appendix C).

**TABLE 4:** Convergence of themes.

Themes	Illustrative/extracted quotation	Article	Similarity rate	Convergence rate
Performance culture conformity	“operational AI applications can significantly reduce caregiver workloads and improve the efficiency of health systems, creating short‐term value that will build positive momentum for more substantial, long‐term investments”	Article 5	After two iterations: similarity rate was 75%	Convergence rate was 80%
	“the value of AI in medical research is measurable, both financially and non‐financially and can drive indicators like the number of projects, access to data, people, and training”	Article 8		
Structural isolation of hierarchical responsibility	“AI companies that respond by simply inserting a human clinician to act as a final checkpoint at the end of the chain risk creating a generation of ‘liability sinks.’ We use the term ‘liability sink’ as an analogy to a ‘heat sink’ in engineering, which absorbs heat from a component. Similarly, in this context, the clinician faces the risk of being used to absorb liability, drawing it away from other parties who also contribute to adverse consequences from the AI system, through their upstream decision‐making beyond the clinician's control”	Article 2	After five iterations: similarity rate was 80%	Convergence rate was 85%
	“a lack of control can also result in a lack of responsibility and accountability … It may become unclear who, why and how a decision was made. The blame of a mistake could for instance be attributed to the developers, implementers, healthcare professional, data supplier and/or system manager of a CDSS”	Article 3		
	“healthcare lawyers interviewed as part of this report, whilst not having seen patients or caregivers bring action against organisations for incorrect diagnoses or treatment linked to AI, were also clear that the accountability ultimately rests with the clinician”	Article 6		
Legitimization through quantification	“AI can help providers integrate leading knowledge and mine health data to find critical signals to prevent patients falling between the cracks and improve adherence to clinical leading practice”	Article 1	After three iterations: similarity rate was 70%	Convergence rate was 90%
	“the ability to extract a large amount of data about an individual's technical performance during a simulated task could be used to provide the trainee with personalized training based on their particular struggles and weaknesses, ultimately eliminating the negative effects of both implicit and explicit bias”	Article 4		
	“one of the strengths of AI in medicine is the ability of AI‐based systems to rapidly process thousands more data points than a person ever could… Once AI systems can learn, they can store and process much more data than can the brain of a physician who sees on average 30 patients a day”	Article 7		

## 5 Discussion

This research makes three contributions to risk management in AI‐enabled healthcare. First, it reframes “*agentic loafing*” from an individual cognitive tendency to a systemic organizational hazard, demonstrating that the most insidious risk is not technical failure but the gradual erosion of human judgment through institutional pressures. Second, it challenges classical delegation theory by showing that AI delegation thrives without monitoring, punishment, or shared objectives; thus, replacing “*credible commitment*” with “*credible disavowal*” and producing what this research terms “*risk evaporation”*: the systematic disappearance of accountability when AI‐assisted decisions fail. Third, it provides a “*preliminary*” but practical diagnostic tool that transforms this hidden risk into a measurable score, accompanied by three intersectional interventions that disrupt the couplings between the institutional drivers of “*agentic loafing*.”

### 5.1 Contextual Contribution

This research built on and extended the concepts of “*social loafing*” and “*algorithmic loafing*” (Inuwa‐Dutse et al. 2023), reframing them as a decision delegation risk in healthcare contexts; thus, introduced a novel phenomenon referred to as “*agentic loafing*.” While prior work explored the concept of “*loafing*” primarily in human–AI collaboration and transparency settings, this research characterized its manifestation in healthcare through three emerging themes: *performance culture conformity*, *structural isolation of hierarchical responsibility*, and *legitimization through quantification*. By situating “*agentic loafing*” within institutional dynamics, this research attempted to advance its theoretical scope beyond individual cognitive tendencies to systemic organizational pressures. By doing so, this research addressed limited empirical research on the central factors influencing delegation decisions in technology use and introduces new antecedents to the concept (Strunk et al. 2024).

### 5.2 Theoretical Contribution

Classical delegation theory (Cooter and Gilbert 2022) is built on a simple trade‐off: delegation saves the principal's time and effort but risks the agent using discretion for its own purposes rather than the principal's. This framework assumes that principals can monitor agents by observing outcomes, punish them when they stray (through firing, budget cuts, or reputation damage), and delegate more when agents share their objectives (ally principle). In government, for example, Congress delegates to agencies because it can hold hearings, cut funding, or rewrite statutes. In courts, appellate judges delegate fact‐finding to trial judges because they can reverse decisions or remand cases. These mechanisms work because human agents are visible, accountable, and responsive to sanctions. However, this research challenges each of these assumptions when the agent is an AI system in healthcare.

In AI‐mediated healthcare, delegation does not just risk diversion of resources; it induces “*agentic loafing*,” which manifests as a systematic uncritical acceptance of algorithmic outputs. Unlike human agents, AI systems cannot be meaningfully monitored because their decision logic is opaque (the “*black box*” problem). They cannot be punished because they cannot be fired, sued, or held professionally accountable. And they have no objectives to share with the principal; an AI does not care about patient outcomes, organizational mission, or clinical judgment, it optimizes for statistical accuracy on its training data, and the evidence of AI's unpredictable decisions in healthcare (Issa et al. 2024) compounds this fundamental misalignment. Paradoxically, the absence of these classical safeguards does not reduce delegation but enables it. When an AI recommends treatment, clinicians can accept it without scrutiny, knowing that if the outcome is poor, they can blame the algorithm. If the outcome is good, they can take credit. In a sense, where classical delegation theory assumed that “*credible commitment*” makes delegation work (Nou 2017), this research reveals that in AI delegation, “*credible disavowal*” makes delegation easier, a risk that classical theory never anticipated.

In a sense, this delegation inversion, from credible commitment to credible disavowal, is not merely a theoretical curiosity. It has observable, measurable consequences for how risk is distributed in AI‐mediated healthcare. When clinicians can blame algorithms for bad outcomes and credit themselves for good ones, the traditional risk‐bearing function of delegation collapses. In classical delegation, the principal bears residual risk (i.e., degree of safety risk; Ewertowski et al. 2024) because the agent can shirk or act opportunistically; the principal monitors, punishes, or withholds delegation accordingly. In AI delegation, however, no party bears residual risk in any meaningful sense. The AI bears no risk because it has no legal personhood. The clinician bears no risk because they can disavow the AI's recommendation. The organization bears risk only insofar as it is named in a lawsuit, but accountability is diffused across developers, implementers, and users. Thus, the result is not risk transfer but “*risk evaporation*,” a systematic disappearance of accountability that leaves no one responsible when AI‐assisted decisions fail, as argued by this research. In this vein, this risk evaporation is the hidden logic of “*agentic loafing*”: clinicians delegate not because they trust the AI, but because they trust that no one will be held accountable if the AI is wrong.

### 5.3 Practical Contribution

**FIGURE 4:**
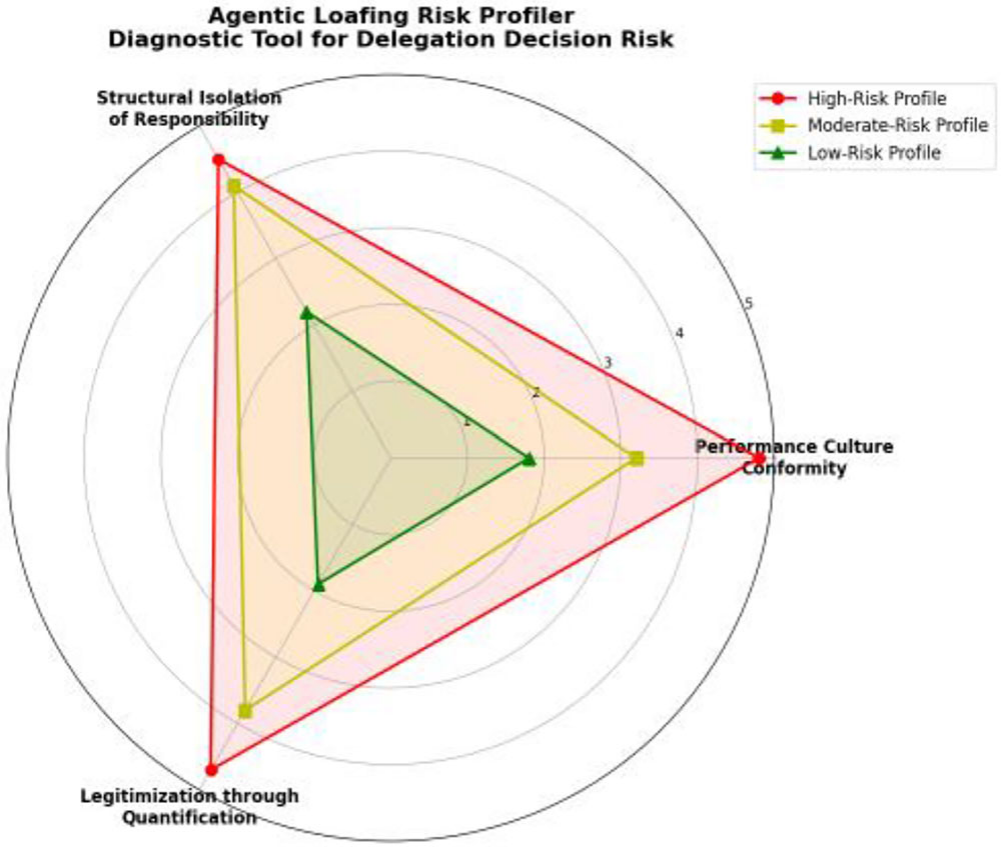
Diagnostic tool as a risk profiler.

Decision delegation has been perceived and explained not as a cost or benefit but rather as a risky bet (Lubars and Tan 2019). Therefore, this research develops a “*preliminary*” diagnostic tool that allows organizations to plot and calibrate “*agentic loafing*” (see Figure 4); thereby, turning the bet into a rational and calculated decision. By doing so, this research addresses calls for proposing evaluation metrics for the loafing phenomenon in AI contexts (Eichholz 2025; Inuwa‐Dutse et al. 2023); thus, setting appropriate delegation boundaries (Rosenberg et al. 2025; Stødle et al. 2025). In a sense, the significance of this diagnostic tool in healthcare is paramount, as it transforms the abstract risk of “*agentic loafing*” into a tangible and quantifiable score that can be tracked, compared, and managed.

**FIGURE D1:**
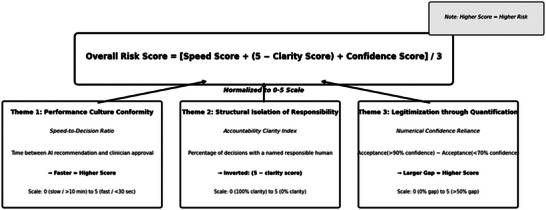
Agentic loafing risk score.

To enable replicable application across different healthcare settings, each of the three dimensions is operationalized using a single, measurable metric. Performance culture conformity is assessed via the “*speed‐to‐decision ratio*” (Statsenko et al. 2021), the average time between AI recommendation and clinician approval, with faster times indicating higher conformity risk. Structural isolation of responsibility is measured using the “*accountability clarity index*” (Nuñez et al. 2016), the percentage of AI‐assisted decisions where a single, named human can be identified as ultimately responsible, with lower clarity indicating higher isolation risk. Legitimization through quantification is captured by the “*numerical confidence reliance gap*” (Fregosi et al. 2026), the difference in clinician acceptance rates between high‐confidence (> 90%) and low‐confidence (< 70%) AI recommendations, with wider gaps indicating greater reliance on quantified outputs. These three metrics are combined into a composite “*Overall Agentic Loafing Risk Score*” ranging from 0 (low risk) to 5 (critical risk), calculated as the arithmetic mean of the three normalized dimension scores (see Figure D1).

**FIGURE D2:**
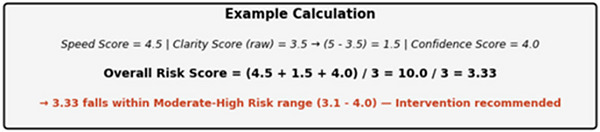
Example on agentic loafing risk score.

For instance, a clinical unit exhibits a speed‐to‐decision ratio score of 4.5 (indicating very fast approval times, approaching < 30 s), an accountability clarity index raw score of 3.5 which inverts to 1.5 (indicating moderately low accountability clarity, approximately 30%–40% of decisions have a named responsible human), and a numerical confidence reliance score of 4.0 (indicating a large gap of approximately 40%–50% between acceptance of high‐confidence vs. low‐confidence AI recommendations) (see Figure D2). Thus, applying the equation, the score falls within the moderate‐high risk range (3.1–4.0), indicating that the unit requires immediate intervention to address “*agentic loafing*.” The score also reveals that the primary driver of risk is numerical confidence reliance (4.0), suggesting that interventions should prioritize reducing overreliance on AI confidence scores before addressing speed or accountability gaps.

**FIGURE D3:**
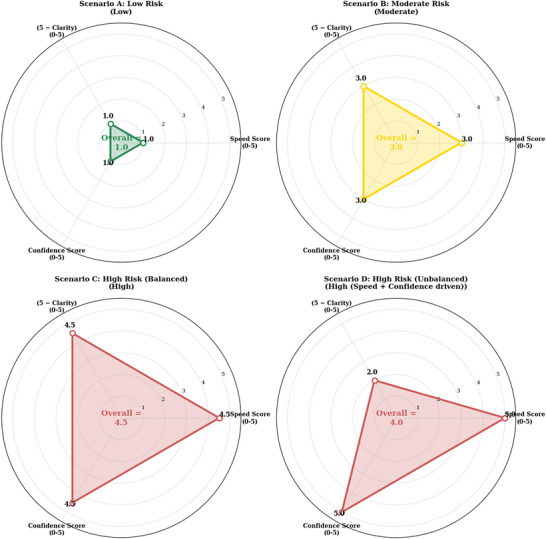
Hypothetical scenarios simulations.

Furthermore, to demonstrate how different metric configurations produce distinct risk profiles, a simulation was conducted across four hypothetical scenarios (see Figure D3). Scenario A assumes all three metrics at 1.0, yielding an overall score of 1.0, represented as a small equilateral triangle on the radar chart, indicating low risk and safe delegation practices. Scenario B assumes all metrics at 3.0, yielding 3.0, a medium equilateral triangle indicating moderate risk requiring active monitoring. Scenario C assumes all metrics at 4.5, yielding 4.5, a large equilateral triangle indicating critical risk across all dimensions. Scenario D assumes speed at 5.0, clarity at 2.0, and confidence at 5.0, yielding 4.0, an elongated radar shape revealing that risk is driven primarily by speed and confidence reliance rather than accountability. In this sense, the radar shape visually diagnoses which dimensions drive “*agentic loafing*”; thus, enabling targeted rather than blanket interventions.

#### 5.3.1 Intersectional Interventions

**FIGURE 5:**
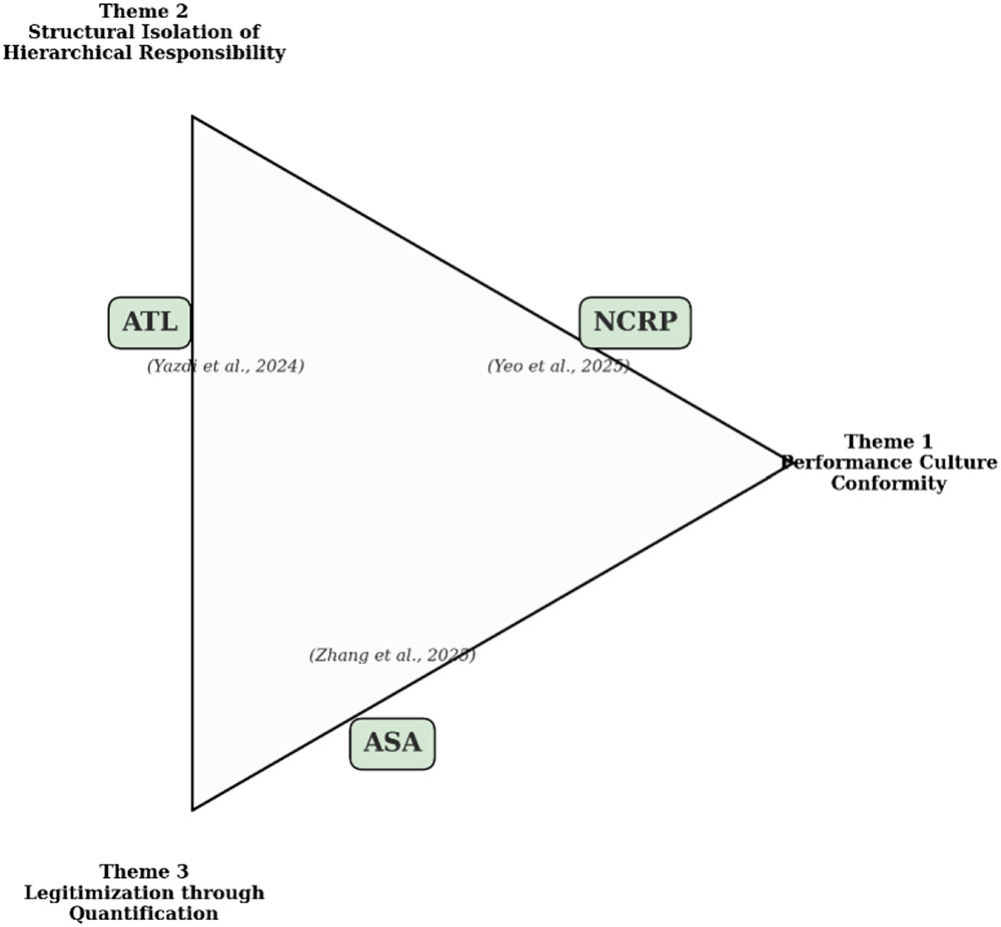
Intersectional interventions.

As evident from the findings, “*agentic loafing*” does not arise from any single driver in isolation but from their mutual reinforcement. Consequently, interventions that target only one dimension will fail because the remaining drivers continue to reinforce delegation behavior. As such, this research argues that effective risk mitigation requires “*intersectional interventions*” that disrupt the coupling between drivers. The three interventions proposed target precisely these couplings: Normative Control Reduction Protocol (NCRP), Accountability Tracing Log (ATL), and Accuracy Standard Auditing (ASA) (see Figure 5).

In a sense, NCRP targets the coupling between performance culture conformity and structural isolation of responsibility (Theme 1–Theme 2). Drawing on cross‐cultural research demonstrating that heightened situational strength manifests as social isolation and workplace bullying in conformity‐oriented cultures (Yeo et al. 2025), this intervention calls for organizations to systematically reduce excessive normative controls. A “*normative control audit*” identifies rules, expectations, and monitoring practices that force silent compliance without accountability. These are replaced with autonomy‐enhancing mechanisms: clear individual decision rights, documented second‐opinion pathways, and protected time for clinicians to question AI recommendations without fear of penalty. By reducing the “*situational strength*” that drives conformity (Theme 1) and prevents peer support (Theme 2), this intervention disrupts the social–psychological mechanism through which “*agentic loafing*” erodes both judgment and belonging.

Also, the coupling between structural isolation of responsibility and legitimization through quantification (Theme 2–Theme 3) is addressed through an ATL. The need for such an intervention is underscored by risk management research showing that accountability becomes complex when decisions are delegated to AI (Yazdi et al. 2024). The log requires three elements for every AI‐assisted decision: the AI's recommendation and confidence score, the clinician's final decision, and the clinician's identity. Monthly random audits of 5% of logs identify patterns of overreliance or diffusion without punitive consequences. By making delegation visible and traceable, this low‐burden intervention restores accountability where AI delegation has rendered it complex and opaque.

Finally, ASA targets the coupling between performance culture conformity and legitimization through quantification (Theme 1–Theme 3). Drawing on research documenting how the relentless pursuit of technical accuracy leads workers to internalize and conform to arbitrary accuracy standards without questioning their legitimacy (Zhang et al. 2025), this intervention requires organizations to periodically audit the accuracy thresholds imposed on AI‐assisted decisions. The audit assesses whether required accuracy levels (e.g., 95% confidence scores) are justified by clinical necessity or reflect arbitrary quantification bias. When thresholds exceed reasonable clinical needs, they must be recalibrated. The audit also mandates transparency: clinicians must be informed of the rationale behind accuracy standards and empowered to question them without penalty. By exposing the arbitrariness behind seemingly objective numbers, this intervention disrupts the blind trust in quantification (Theme 3) that drives uncritical conformity (Theme 1).

## 6 Limitations and Future Research Directions

Two main limitations exist, which pertain to the practical dimension of this research rather than its theoretical or methodological contributions. First, each metric reduces a complex, multidimensional construct to a single quantitative proxy. For instance, the speed‐to‐decision ratio captures temporal efficiency but overlooks other conformity behaviors such as reduced information seeking or diminished error detection. Similarly, the accountability clarity index assumes that naming a responsible human equates to functional accountability, yet in practice, named individuals may lack authority, resources, or meaningful oversight over AI‐generated decisions. Second, the equal weighting of the three metrics in the composite score assumes they contribute equally to “*agentic loafing*” risk, which is an assumption that remains theoretically plausible but empirically untested. Therefore, future research should validate these metrics through longitudinal studies in active clinical AI deployments, explore differential weighting schemes based on empirical risk prediction, and develop complementary qualitative measures to capture the full behavioral spectrum of “*agentic loafing*.”

## 7 Conclusion

In conclusion, this research reveals “*agentic loafing*” as an unrecognized risk in AI‐enabled healthcare, one that has quietly evolved from social to algorithmic to agentic forms. Unlike technical failures, which announce themselves through visible malfunction, this risk hides within routine workflows, eroding judgment and accountability long before any visible failure occurs. The value of this research, therefore, lies in making this hidden risk visible: first, by identifying its three institutional drivers; second, by providing risk managers with a “*preliminary*” diagnostic tool to detect and measure the appropriate risk score. From this, a critical deduction follows: the most dangerous AI systems are not those that “*fail unpredictably*” but those that function just “*reliably enough*” to quietly erode human judgment without ever triggering an alarm. For the field of risk analysis, this implies that the most consequential AI risks may not be technical at all, but behavioral and institutional; hence, demanding new forms of risk governance that look beyond algorithms to the human conditions that silently enable decision delegation to go unchecked.

## Conflicts of Interest

The authors declare no conflicts of interest.

## Figures and Tables

**FIGURE A1 risa70306-fig-0006:**
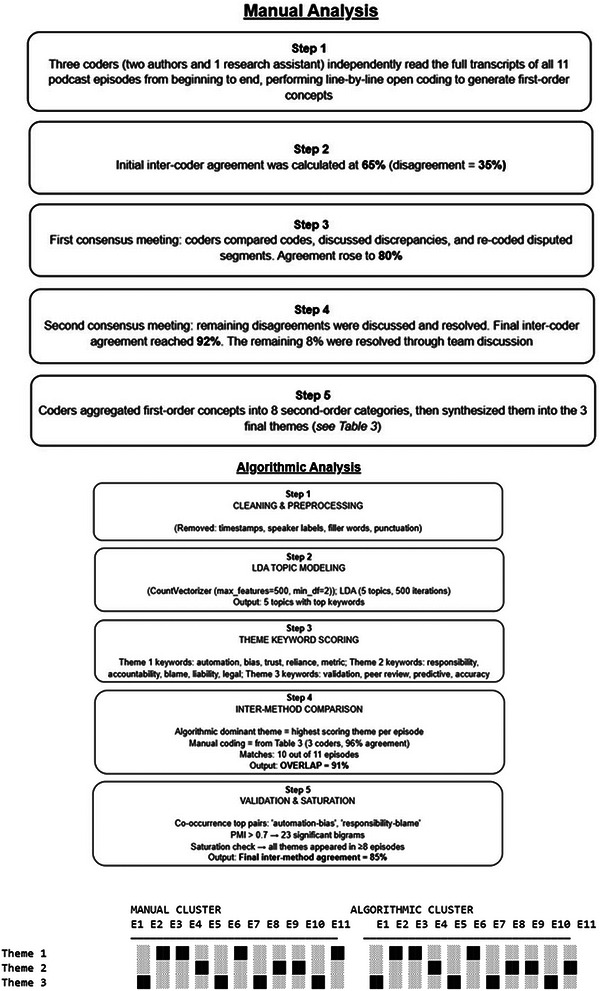
Manual versus algorithmic cluster comparison.

**FIGURE B1 risa70306-fig-0007:**
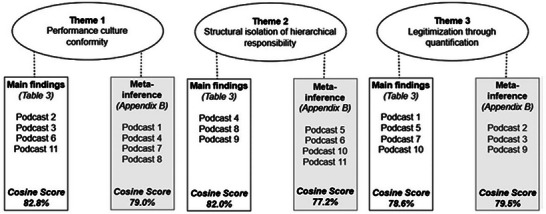
Meta‐inference association map.

## Data Availability

Data are available upon request.

## Appendix A

### A.1

 [Fig risa70306-fig-0006]

## Appendix B

### B.1

**Table:**

Themes	Second‐order concepts/categories	Illustrative quotations from the podcasts episodes	Source
Performance culture conformity	Systemic prioritization of expediency over scrutiny	“We're working on hypertension. So these happen to be around improving blood pressure screening or, you know, just some of the, you know, kind of key quality metrics associated with seeing those patients”	Podcast episode 1
		“The driving force behind a lot of this, as the expert pointed out, is freeing up clinicians, speeding up those back end administrative tasks. Notes. Scheduling. Managing appointments allows doctors and nurses to spend more valuable time directly with patients”	Podcast episode 4
		“AI helps predict volumes so you can staff appropriately. The business of medicine needs to refine maximize documentation to build to the highest level of acuity”	Podcast episode 7
		“There are a number of practices across the country who are now using digital scribes… A physician may have an interaction with a patient, have a visit with a patient, and then at the end of the meeting, the digital scribe will spit out a draft note”	Podcast episode 8
Structural isolation of hierarchical responsibility	Fundamental dissolution of actionable accountability	“Summarization very quickly and really extract the information that's relevant, send it to the emergency managers. But accountability is again critical”	Podcast episode 5
		“We created three parallel teams, a model development team, an implementation team, and an evaluation team… their work was very overlap”	Podcast episode 6
		“Vendors building complicated models… it's really incumbent upon them to let us know what were the training data? Where did these large language models come from?”	Podcast episode 10
		“My biggest worry with using any of these models, whether it be like a AI machine learning algorithm…is that still feels very that is part of the institution…Like, why should I trust, especially an AI black box model that could be easily manipulated?”	Podcast episode 11
Legitimization through quantification	Supremacy of metricized authority	“The algorithm performed as well or better than 21 board‐certified dermatologists. The area under the ROC curve was 0.94”	Podcast episode 2
		“Explainability methods can make a model less fair… they can increase overreliance sometimes even because, again, if you just have a number or if you just have a description risk of violence, it doesn't really short circuit that critical thinking that you have to do to make the decision”	Podcast episode 3
		“You have very clear KPIs, you know what's your outcome out of here… you have very clear KPIs, you know where it's going”	Podcast episode 9

 [Fig risa70306-fig-0007]

## Appendix C

### C.1

**Table:**

Article	Supporting Literature (Risk management, decision‐making, and healthcare domains)	Significance to the theme
Articles 1, 4, and 7	Wang et al. (2025)—AI uncertainty quantification	Wang et al. (2025) demonstrate that AI systems generate numerical confidence scores, probability distributions, and uncertainty metrics (e.g., predictive entropy, calibration errors) that users interpret as objective measures of reliability. This quantification, whether well‐calibrated or not, creates a psychological anchor that legitimizes AI recommendations, reducing users’ critical scrutiny. In healthcare contexts, such quantified outputs (e.g., 94% confidence in a diagnosis) are often accepted as inherently trustworthy, directly supporting *Theme 3's claim that quantification provides the rationalization for uncritical AI delegation*
Articles 2, 3, and 6	Yin (2026)—Ambiguous responsibility	Yin (2026) systematically demonstrates how the introduction of medical AI diffuses accountability across multiple stakeholders (e.g., manufacturers, medical institutions, clinicians, and regulators) without clearly allocating final responsibility. The paper's dynamic responsibility matrix shows that as AI autonomy increases (from tool‐type to autonomous‐type), accountability becomes increasingly fragmented, creating what is termed as blurred human‐machine boundaries and multifactor causation. This fragmentation enables what the present research calls *structural isolation of responsibility (Theme 2)*, where clinicians can defer to algorithmic outputs while manufacturers claim their systems are only advisory, and institutions point to regulatory gaps, leaving no single party fully accountable when AI‐assisted decisions fail
Articles 5 and 8	van de Wetering and Nguyen (2025)—Performance culture	van de Wetering and Nguyen (2025) examines how hospitals leverage AI‐driven big data analytics to balance exploration (innovation) and exploitation (efficiency). It demonstrates that *performance pressures*, (e.g., improving service metrics, reducing wait times, and meeting quality benchmarks) drive healthcare organizations to *conform to AI‐generated recommendations*. This conformity reflects a culture where algorithmic outputs define “good performance,” leading clinicians and administrators to prioritize measurable efficiency gains over critical scrutiny, *thereby reinforcing the performance culture conformity (Theme 1) driver of agentic loafing*
